# Pancreatic Cancer Biomarkers and Their Implication in Cancer Diagnosis and Epidemiology

**DOI:** 10.3390/cancers2041830

**Published:** 2010-11-02

**Authors:** Mukesh Verma

**Affiliations:** Methods and Technologies Branch, Epidemiology and Genetics Research Program, Division of Cancer Control and Population Sciences, National Cancer Institute, National Institues of Health (NIH), 6130 Executive Blvd., Suite 5100. Bethesda, MD 20892-7324, USA; E-Mail: vermam@mail.nih.gov; Tel.: +1 301 594 7344; Fax: +1-301 435 5477

**Keywords:** biomarker, cancer, diagnosis, epidemiology, epigenetics, glycans, methylation index, pancreas, prognosis, sensitivity, specificity, survival, treatment

## Abstract

Pancreatic cancer is the fourth most common cause of cancer-related mortality in the United States. Biomarkers are needed to detect this cancer early during the disease development and for screening populations to identify those who are at risk. In cancer, “biomarker” refers to a substance or process that is indicative of the presence of cancer in the body. A biomarker might be either a molecule secreted by a tumor or it can be a specific response of the body to the presence of cancer. Genetic, epigenetic, proteomic, glycomic, and imaging biomarkers can be used for cancer diagnosis, prognosis, and epidemiology. A number of potential biomarkers have been identified for pancreatic cancer. These markers can be assayed in non-invasively collected biofluids. These biomarkers need analytical and clinical validation so that they can be used for the purpose of screening and diagnosing pancreatic cancer and determining disease prognosis. In this article, the latest developments in pancreatic cancer biomarkers are discussed.

## 1. Introduction: Pancreatic Cancer Incidence and Mortality

Pancreatic cancer incidence and mortality rates are almost five years in the United States. Due to the difficulty in detecting pancreatic cancer, it is not typically diagnosed until the disease has progressed to advanced stages and treatment options are limited [1,2,3]. Pancreatic cancer is the fourth leading cause of cancer related deaths in the United States and about 38,000 Americans died last year due to this cancer [4,5]. The five year survival rate for individuals with pancreatic cancer is only 5%. Treatment includes surgical resection but 80–90% of patients who undergo this surgery suffer a relapse and die from metastatic or disseminated disease. Sometimes a combination of surgery and chemotherapy is also applied.

The risk factors for pancreatic cancer include smoking, long-standing diabetes and alcoholism, however, hypermethylation of p14 and p16 is now considered a risk factor in K-ras mutated subjects [2,6]. This cancer is strongly associated with the development of hyperglycemia, peripheral insulin resistance and diabetes mellitus, especially when presented as new-onset diabetes mellitus. In a nested case-control study of more than 500 participants, Stolzenberg-Solomon *et al.* demonstrated adeponectin concentrations as risk factors in male smokers [7]. Conditional logistic regression adjusted for smoking, blood pressure, and C-peptide levels was used to calculate odds ratios and 95% confidence intervals for pancreatic cancer in this study.

## 2. Defining Biomarkers

A biomarker is a characteristic that is objectively measured and evaluated as an indicator of normal biologic processes, pathogenic processes, or pharmacologic responses to a therapeutic intervention. Identifying and validating a biomarker which can be used in a clinic setting with high specificity and sensitivity has always been challenging. Pancreatic cancer is a unique cancer where early detection markers have not been identified and validated [1,2,8,9,10]. Sensitivity of a biomarker is its ability to detect those with true cases of pancreatic cancer identified by the clinical criteria. Thus, it is the probability that a person with pancreatic cancer or who will develop the disease will have positive test results. Specificity of a biomarker is a measure of its ability to detect those with true non-cases of disorder identified by the clinical criteria. An ideal biomarker of pancreatic cancer should have high sensitivity and specificity.

## 3. Genetic Biomarkers

RAS is a tyrosine receptor which is involved in multiple cancers and plays a key role in cell growth, transformation and maintenance of tumor phenotype [8]. Oncogenic gain of function mutation in K-ras is a risk factor in pancreatic cancer and used for early detection of the cancer [11,12]. K-ras mutations are also present in lung and colon cancers. Studies were conducted to identify single nucleotide polymorphisms associated with pancreatic cancer but attempts were unsuccessful. Other risk factors (family history, life style, exposure history) should be considered before making conclusions about a pancreatic cancer diagnosis [13].

## 4. Epigenetic Biomarkers

In most studies involving pancreatic cancer, the methylation component of epigenetics has been studied more than histone modifications, miRNA profiles, and chromatin conformational changes [14]. The Methylation Index (MI) of several genes was compared between the malignant and benign groups and genes that were found to be hypermethylated included *DKK3, p16, SFRP2, DKK2, NPTX2* and *ppENK*. Large scale validation of these markers has yet to be completed.

## 5. miRNA Biomarkers

MicroRNAs (miRNAs) are small noncoding transcripts involved in many cellular mechanisms, including tumorigenesis. Recently, attempts have been made to identify pancreatic cancer associated miRNAs and develop methods to determine their quantity at initial and advanced stages of cancer development. miR-21 and miR-196a are among those miRNAs which exhibit differential expression in pancreatic cancer [15,16]. miR-210 plasma levels have been reported to be high in pancreatic cancer patients by Ho *et al.* [17].

## 6. Proteomic Biomarkers

Serum is a good source of biomarkers for detecting pancreatic cancer. Some investigators have performed serum profiling studies and found more serum profiling variability from pancreatic cancer patients than from healthy controls [6,9,18,19,20]. Elevated levels of insulin-like growth factor (IGF) and its receptors have been reported in pancreatic cancer patients [21]. Investigators have attempted to characterize proteins expressed during the pancreatic intraepithelial neoplasias (PanINs) stage which is considered the early stage of pancreatic cancer. In another study, a group of proteins (with a specific protein called anterior gradient 2) have been identified by proteomic approaches of PanINs samples [22,23]. Gao *et al.* have identified serine proteinase-2 (PRSS2) pre-proprotein and pancreatic lipase related protein-1 (PLRP1) from pancreatic juice of pancreatic cancer patients [24]. Cytokeretin 18 was also reported to be a pancreatic cancer marker [25]. Takayama *et al.* reported serum REG4 for pancreatic diagnosis. Stable isotope-labeled proteome (SILAP) standard with extensive multidimensional separation in combination with tandem MS-MS has been used for separating serum samples from pancreatic cancer patients [6]. Few investigators have combined SILAP with iso-electrofocusing (to concentrate proteins by immune-affinity) and 2D LC MS-MS in order to increase specificity of serum biomarkers. This approach gives excellent quantitative results [26]. The upregulation of neutrophil gelatinase-associated lipocalin (NGAL), a 24 kDa glycoprotein, has been reported to be nearly 27-fold in pancreatic cancer cells compared to normal ductal cells in a microarray analysis. Inflammatory-driven processes are involved in pancreatic carcinogenesis because inflammation-sensitive proteins were increased in pancreatic cancer sera. Technologies such as Liquid ESI-MS analyses of sera, used in identifying biomarkers, hold promise for future pancreatic cancer blood tests. The variability observed between the low-mass regions of normal *versus* pancreatic cancer spectra may aid in diagnosis and therapy.

## 8. Other Biomarkers

Messenger RNA (mRNA) expression profiles have been used to detect pancreatic cancer [2]. Imaging techniques also have potential in pancreatic cancer diagnosis, especially in determining the therapeutic response and disease stratification [27]. Splice variants (alternative individual splice sites, alternative exons, alternative introns) have been recommended as pancreatic cancer biomarkers by Hayes *et al.* [28]. Systems biology approaches to identify novel markers associated with pancreatic cancer are also being considered by a few investigators in the field. This approach demands complex statistical modeling. Splice variants either change protein structure or affect gene regulation by changing mRNA stability [29,30]. Generally, splice variants are stable and their high-throughput quantitation is possible [30].

Circulating endothelial cells in serum of patients undergoing treatment have been observed and may serve as biomarkers for response to treatment [31]. Different growth factors can be measured within these endothelial cells by standard protocols. Salivary transcriptomic biomarkers have been identified by Zhang *et al.* which have potential in pancreatic cancer detection and diagnosis [32].

Attempts have been made to isolate pancreatic cancer markers in the stool of patients but the specificity of markers identified has been too low to warrant further investigation [33]. Mucin, *MUC1*, has also been reported to be upregulated and abnormally glycosylated in pancreatic cancer [1,34].

## 9. Biomarkers in Pancreatic Cancer Diagnosis and Epidemiology

Blood has been recognized as a highly important source of disease-related biomarkers [1,3,9,35]. Pancreatic biomarkers have been assayed in biofluids (bile), fine needle tissue aspirate, tissues samples and formalin fixed samples, and stool [13,15,36,37]. Serum REG4 has been extensively studied in epidemiologic studies and seems to be an excellent diagnostics marker [6]. Laiyemo *et al.* conducted a prospective cohort study to identify populations at high risk of pancreatic cancer [38]. Fong *et al.* used a new marker, human trophoblast cell-surface antigen (TROP2), to evaluate its correlation with aggressiveness and prognosis of pancreatic cancer [39]. They applied immunohistochemistry technology in paraffin-embedded primary tumor tissue samples from a series of consecutive patients with pancreatic adenocarcinoma. TROP2 was significantly associated with decreased overall survival in this study.

## 10. Challenges, Potential Solutions, and Future Perspective

The development of effective tools for the early detection of pancreatic cancer, or its precursors, in high-risk subjects could play a key role in reducing the burden of this disease, which is the most lethal among solid gastrointestinal tumors [1]. It is difficult to access the pancreas due to its anatomical location and imaging techniques are also difficult to apply for observation of deformities of the organ during disease development. Biomarkers discussed above may be considered for further investigation in large prospective cohort studies and pooled analyses with other prospective cohorts. One of the biggest challenges has been the observation of a few proteins present in large abundance which are not related with the disease and the protein biomarker is present in low concentrations. Specific removal of those proteins has been difficult although a few promising approaches, called isobaric tags for relative and absolute quantification, or iTRAQ, have been developed [26,35].

Pancreatic ductal adenocarcinoma has a tendency to recur and adjuvant therapy is recommended after surgical resection [15]. It has also been suggested that biomarkers which could predict treatment outcome would be extremely useful. miRNAs may be appropriate for this purpose. miR-21 turns out to be a marker with promise because its level decreases with successful therapy. There is still a need for additional novel biomarkers which could be used for pancreatic cancer prognosis and response to therapy. Some promise appears with gemcitabin-based chemotherapy and utilization of cyclo-oxygenase-2 (Cox-2) and vascular endothelial growth factor (VEGF), which are regulated by an mRNA binding protein called Hu protein antigen R or HuR, as therapy response markers [5,40]. HuR binds to VEGF mRNA and alters VEGF expression. Such an approach has potential application in personalized medicine. Alternative therapeutic approaches using inhibitors are also promising [41,42]. Biomarkers which distinguish pancreatitis from pancreatic cancer also should be further characterized.

For all biomarkers, especially genetic biomarkers, the guidelines developed by the Evaluation of Genomic Applications in Practice and Prevention (EGAPP) Initiative, established by the National Office of Public Health Genomics at the Centers for Disease Control and Prevention, should be followed [43]. EGAPP emphasizes that each marker must pass the test of analytic validity, clinical validity, and clinical utility as defined by the EGAPP Working Group (EWG). These guidelines support the development and implementation of a rigorous, evidence-based process for evaluating genetic tests and other genomic applications for clinical and public health practice in the United States.

Multiplexing and use of multiple markers (genetic, epigenetic, and proteomic) in the same sample may increase the sensitivity and specificity of biomarkers in diagnosis but validation of such an approach has not been accomplished yet and should be considered. Although an abnormal tumor marker level may suggest cancer, this alone is usually not enough to diagnose cancer. Therefore, measurements of tumor markers are usually combined with biopsy results to diagnose cancer. Furthermore, patient related information (family history, diet and life style, behavior) helps tremendously in the accurate diagnosis of cancer. Finally, we are still waiting for a group of biomarkers or a specific biomarker to identify patients with asymptomatic pancreatic cancer. This needs collaboration of epidemiologists, population scientists, basic and clinical scientists, industry and funding agencies.
